# Macromolecular Hydrodynamics and Fractal Structures of the Lignins of Fir Wood and Oat Husks

**DOI:** 10.3390/polym15173624

**Published:** 2023-09-01

**Authors:** Anatoly Karmanov, Lyudmila Kocheva, Mikhail Borisenkov, Vladimir Belyi

**Affiliations:** 1 Institute of Biology of Komi Science Centre of the Ural Branch of the Russian Academy of Sciences, Syktyvkar 167000, Russia; apk0948@yandex.ru; 2 Institute of Geology of Komi Science Centre of the Ural Branch of the Russian Academy of Sciences, Syktyvkar 167000, Russia; 3 Institute of Physiology of Komi Science Centre of the Ural Branch of the Russian Academy of Sciences, Syktyvkar 167000, Russia; 4 Institute of Chemistry of Komi Science Centre of the Ural Branch of the Russian Academy of Sciences, Syktyvkar 167000, Russia

**Keywords:** lignin, macromolecular topology, hydrodynamic properties, fractal dimension

## Abstract

The topological structure of the macromolecules of lignins isolated from oat husk and fir wood was studied by means of macromolecular hydrodynamic methods. The macromolecular properties were analyzed by evaluating the intrinsic viscosity and coefficients of the translational diffusion and the sedimentation velocity of the lignins in dilute dimethylformamide solutions. The average molecular weights (M_Dη_) and polydispersity parameters were calculated based on the results of the fractionation, as follows: M_w_ = 14.6 × 10^3^, M_n_ = 9.0, and M_w_/M_n_ = 1.62 for lignins from fir wood and M_w_ = 14.9 M_n_ = 13.5 and M_w_/M_n_ = 1.1 for lignins from oat husks. The fractal analysis of the lignin macromolecules allowed us to identify the distinctive characteristics of the fractal and topological structures of these lignins. The measurements indicated that the fractal dimension (d_f_) values of the guaiacyl-syringyl lignins from oat husks were between 1.71 and 1.85, while the d_f_ of a typical guaiacyl lignin from fir wood was ~2.3. Thus, we determined that the lignin macromolecules of oat husks belong to the diffusion-limited aggregation-type cluster–cluster class of fractals of the Meakin–Kolb type, with a predominance of characteristics common to a linear configuration. The lignins of softwood fir trees exhibited a branched topological structure, and they belong to the diffusion-limited aggregation-type particle–cluster class of fractals of the Witten–Sander type. Lignins from oat husks have the linear topology of macromolecules while the macromolecules of the lignins from fir wood can be characterized as highly branched polymers.

## 1. Introduction

Lignins are polyfunctional biopolymers synthesized by higher plants [1]. They are part of the xylem of all woody plants, both deciduous and coniferous. It should be noted that lignins are present in different quantities both in sapwood and heartwood, both in earlywood and latewood [2]. Lignins were found in all vegetative and even generative organs in plants. Lignins are present in the shells of nuts and cereal grains and, in particular, in oat husks [3,4,5].

Scientists have been searching for the effective and competent use of natural and industrial lignins for many decades, but to date, the problem of the valorization of these polymeric compounds remains largely unresolved [6]. It should be noted that lignins have a number of advantages over other biopolymers, including: (1) an almost inexhaustible raw material base; (2) lignins are part of many examples of plant-based foods, which indicates their safety; and (3) lignins of various botanical origins differ in their chemical and topological macromolecule structures. These features of lignins point to the potential possibility of creating a wide range of practically useful products with different structures and desired properties [7].

An analysis of the literature indicates that lignins, which are part of plant products, including oats, have a high physiological activity [8]. Oat lignins have very high antioxidant properties, quite comparable with those of medical antioxidants such as mitofen [9]. Data have been obtained showing that lignins have a positive effect on the mechanism of the hepato-enteric circulation of such sex hormones as estradiol, estrone, and progesterone [9,10]. The ability to regulate the level of sex steroid hormones with the help of lignin preparations can help prevent cancers in human reproductive organs [10].

It should be noted that future advances in the use of lignins require fundamental studies of both the chemical and topological structures of this biopolymer. To continue the development of the physicochemistry of lignins as typical macromolecular compounds, it becomes necessary to introduce an additional criterium: the fractal dimension [11,12,13], which will make it possible to identify the topological structures of lignins more reliably. Knowledge of the topology of any polymer, including a lignin, makes it possible to predict the physical and physicochemical properties of materials based on, for example, fiber-forming or adsorption properties with respect to various toxicants, such as mycotoxins and radionuclides [14,15].

The concept of lignin structure continues to change; for example, earlier, lignins were mostly considered as network polymers, but now, some researchers have described lignins as low molecular weight polymers with a linear structure [16,17]. According to other researchers, lignins are polydisperse polymers containing many polymer chains with various lengths and branching. As a result, a lignin macromolecule can be considered as a complex structure with a hyperbranched topology [18,19,20].

According to results presented in the literature [21,22,23], softwood lignins are randomly branched polymers. The topological and fractal structures of lignins in non-woody plants have been seldom studied. It should be emphasized that most of the conclusions about the topologies of lignin macromolecules that have been made in recent years were based primarily on the results of chemical studies and not on classical transport methods for studying the topological structures of macromolecules.

Thus, the question of the topologies of lignin macromolecules remains the subject of discussion. In addition, the problem of the topotaxonomic classification of natural lignins remains unresolved. Therefore, new studies are required using the following classical research methods: capillary viscometry, sedimentation velocity, and translational diffusion [24,25]. These methods allow the direct determination of the coefficients of the rotational and translational friction of molecules. As a result, these make it possible to reliably determine the dimensions and conformational properties of polymers and, ultimately, the topological structures of macromolecules. The importance and relevance of knowledge about the topologies of macromolecules can hardly be overestimated since the configurations of macromolecules determine the sizes of lignin macromolecules in solutions and, accordingly, the properties and reactivity levels of lignins in various fields of biomedical use.

The least studied class of lignin polymers is monocotyledonous angiosperms. Studies on the lignins of herbaceous plants, in particular, cereals, are very relevant from the point of view of creating new biodegradable materials with desired properties for use in medicine [9]. Detailed studies of the macromolecular properties of lignins of various biological origins can help to get closer to the solution of the problem of lignin application [26].

This work is devoted to the quantitative assessment of the hydrodynamic, transport, and fractal characteristics of oat lignin macromolecules in dilute solutions by the classical methods of polymer physicochemistry in order to establish a model type of topology. The parallel comparative study of fir lignin, as the most studied polymer, makes it possible to more objectively assess the structural differences in the topological structures of lignin macromolecules of various biological (taxonomic) origin.

## 2. Materials and Methods

The lignin samples were isolated from oat (*Avena sativa* L.) husks (Sysol’sk Variety Test Station, Vizinga, Komi Republic, Russia) and wood from the trunk of a fir tree (*Abies sibirica* Ledeb.) (Syktyvdinsky District, Syktyvkar, Komi Republic, Russia). The ages of the trees were ~35 years. The wood samples of the trees from the same forest stand were collected at a height of 2 m.

Both plant samples were ground to a particle size of 0.4 mm using a laboratory mill, extracted successively with ethanol-benzene and water, and dried at room temperature. The two samples of lignins were obtained by the dioxane method, according to the following method used by Pepper [27]: the lignins from oat husks (OHL) and the lignins from the fir wood (FWL) were determined to have yields of 41% and 19%, respectively, according to the Klason lignin contents, and this was pre-determined in two repetitions for the samples of the extracted wood meal [28] of the raw plant materials. The solvents used were N,N-dimethylformamide (DMF), (T_boiling_ = 152.5 °C, ρ_o_^25^ = 0.9473 g/cm^3^, η_o_^25^ = 0.78×10^−2^ Pa·s, and n_D_^20^ = 1.4305; «Component-Reactiv», Moscow, Russia); dioxane (T_boiling_ = 101.3 °C and ρ_o_^25^ = 1.0336 g/cm^3^; «Component-Reactiv», Moscow, Russia); and benzene (T_boiling_ = 80.1 °C and ρ_o_^25^ = 0.878 g/cm^3^; «Component-Reactiv», Moscow, Russia).

The elemental analyses of the lignins were carried out using an 195 EA 1110-CHNS-O elemental analyzer (CE Instruments, Wigan, UK). The phenolic (PhOH) and carboxylic (COOH) groups were determined using the chemisorption method [29].

The FTIR spectra of the lignin samples were obtained with an IR-Fourier spectrometer IFS 25 (Bruker, Billerica, MA, USA), and they had the following major absorption bands: OHL: 3440, 1717, 1595, 1515, 1465, 1422, 1256, 1163, 1121, 1032, and 839 cm^−1^ and FWL: 3440, 1720, 1610, 1520, 1470, 1430, 1365, 1335, 1270, 1130, 1035, and 840 cm^−1^.

The ^13^C NMR spectra (75.5 MHz) of the OHL and FWL were recorded on a Bruker AM 300 spectrometer (Bruker, Billerica, MA, USA). Deuterated dimethylformamide (DMF-d7) was used as a solvent. The chemical shifts (δ in ppm) were as follows: OHL: 15.5, 18.8, 23.4, 24.9, 29.4, 34.1, 56.0, 60.5, 65.4, 66.8, 72.1, 77.1, 77.6, 80.1, 82.9, 94.8, 96.5, 99.4, 102.4, 104.3, 105.2, 111.3, 115.1, 115.8, 116.3, 119.5, 121.4, 125.5, 128.6, 129.5, 130.7, and 133.4 and FWL: 28.8, 53.3, 53.7, 55.6, 60.1, 62.7, 63.0, 63.2, 65.2, 65.6, 71.2, 71.7, 83.2, 86.6, 111.0, 111.4, 112.7, 114.7, 115.0, 118.4, 119.9, 122.8, 125.9, 128.0, 129.3, 131.1, 132.0, 133.0, 134.6, 143.3, 145.1, 145.8, 146.8, 166.8, and 171.6.

The lignins were fractionated by the fractional precipitation from a dioxane solution to benzene (the precipitating agent). The methods of viscometry, isothermal translational diffusion, and sedimentation velocity were used to investigate the hydrodynamic properties of the lignin macromolecules [30].

The viscometric analyses were carried out at 298 K by means of an Ostwald viscometer (the radius of the capillary was 5.6 × 10^−4^ m). The intrinsic viscosities ([η]) of the polymer fractions were determined by the linear extrapolation of the [η]_sp_/c dependencies to an infinite dilution. The experimentally determined relative viscosity values and the Huggins Equation (1) were used to calculate the value of [η]:

η_sp_/c = [η_c_] + k_H_[η]_c_^2^c +…, (1)

where c is a mass concentration, k_H_ is the Huggins coefficient, [η] = [η]ρ_0_, and ρ_0_ is the solvent density.

For the translational diffusion analysis, the diffusion coefficients (D) were determined with an MOM 3180 ultracentrifuge (MOM Hungarian Optical Works, Budapest, Hungary) using a polyamide boundary forming cell. The rotor speed was 5 × 10^3^ rpm. The measurements were carried out using diluted solutions (c ≤ 3 × 10^−3^ g/cm^3^). The diffusion was measured via optical recordings of the distribution of macromolecules (∂c/∂x) according to the shifts (x) in the solution–solvent interface. The diffusion curve (the distribution δ(x)) was determined using Equation (2):

δ(x) ~ ∂n /∂x = (dn/dc)∂c/∂x,(2)

where (dn/dc) is the refractive index increment of the polymer–solvent system and (dn/dc) = lim(n − n_0_)/c, where n is the refractive index of the solution with the concentration c and n_0_ is the refractive index of the solvent.

The diffusion coefficient D was calculated from the experimental data using Equation (3):

(4πDt)^0.5^ = S/H, (3)

where S is the area under an interference fringe and H is the maximum ordinate.

The coefficients (D) were calculated according to the following sequence:

1. determination of the maximum ordinate (H) of the diffusion curve (mm)
2. measurement of the area under the curve (S) (mm^2^)
3. determination of the S/H values and the argument (x)
4. construction of the dependence of the diffusion boundary dispersion (S/H)^2^ on t

The slope of this dependence was used to find the value of the diffusion coefficient, D, as follows: D = (1/2)∂(S/H)^2^*/*∂t.

For the sedimentation analysis, the sedimentation coefficients (s) were measured using an analytical ultracentrifuge (an MOM-3180) in a double-sector polyamide cell, allowing for the formation of an artificial boundary at 48 × 10^3^ rpm. The experiments implied measuring the rate of the maximum sedimentogram ordinate shift. The solution concentrations varied in the range of 0.001 ˂ c ˂ 0.002 g/cm^3^. The sedimentation coefficients were calculated as follows (Equation (4)):

s = (Δlnx/Δt)ω^−2^, (4)

where ω = 2πn is the rotation frequency of the centrifuge rotor and x is the maximum coordinate in the sedimentogram.

Since the Debye criterion was very low in the experiments (c[η] ˂ 0.025), the influence of the concentration on the value of the sedimentation coefficient (s) was omitted.

For calculation of the molecular masses (MWs), the high-molecular-weight fractions were calculated using the values of D and s and the buoyancy factor in accordance with the Svedberg equation. The values (M_sD_) of each fraction were calculated using the corresponding experimental data (Equation (5)):

(5) $$M_{\mathrm{sD}}=\mathrm{sRT}/(1-\bar{\nu }\rho_{o})D,$$

where $\left(1-\bar{\nu }\rho_{o}\right)$ is the buoyancy factor, and its values were 0.329 for the sample OHL and 0.336 for FWL.

For the low-molecular-weight fractions, the M_Dη_ values were determined using the following (Equation (6)):

M_Dη_ = A_o_^3^([D]^3^[η]), (6)

where [D] = η_0_DT and A_0_ are the Tsvetkov–Klenin hydrodynamic invariants determined from the results of the sedimentation–diffusion analysis of the high-molecular-weight fractions, and they were determined using Equation (7):

A_0_ = η_0_D(M_sD_[η])^1/3^/T. (7)

The average M_Dη_ molecular weights and polydispersity parameters of the samples were calculated based on the results of the following fractionation: M_w_ = 14.6 × 10^3^, M_n_ = 9.0, and M_w_/M_n_ = 1.62 for the FWL and M_w_ = 14.9, M_n_ = 13.5, and M_w_/M_n_ = 1.1 for the OHL. The mathematical analyses of the experimental data, including the calculation of the root mean square errors, were performed using the Origin 6.1 software package.

## 3. Results and Discussion

The results of the ^13^C NMR, FTIR-spectroscopy, and chemical analyses showed that the samples belonged to different classes of lignins, as follows: the FWLs were typical guaiacyl lignins and the OHLs belonged to the guaiacyl-syringyl class of lignins. The elemental content of the OHLs was 60.5% (C), 5.3% (H), and 34.2% (O). The elemental content of the FWLs was 65.1% (C), 6.3% (H), and 28.6% (O). The methoxy group contents were 16.1% (OHLs) and 14.4% (FWLs). The phenolic (PhOH) and carboxylic (COOH) group contents were PhOH: 6.6% (OHLs) and 2.3% (FWLs) and COOH: 4.3% (OHLs) and 3.2% (FWLs). The averaged monomeric unit formulas were calculated using the elemental analyses and the contents of the methoxy groups, as follows: C_9_H_7.39_O_3.21_(OCH_3_)_1.05_ for the OHLs and C_9_H_9.2_O_2.8_(OCH_3_)_0.93_ for the FWLs.

The topological structures of the macromolecules and the features of the macromolecular coils as fractal objects [11,31] could be established by analyzing their hydrodynamic properties, along with their macromolecular sizes and/or weights.

**Figure 1:**
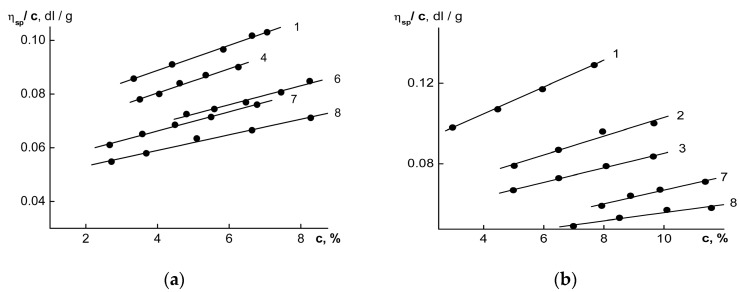
The dependencies of the reduced viscosities η_sp_/c on the concentrations of the solutions of the OHL (**a**) and FWL (**b**) fractions. The numbers of the lines correspond to the numbers of the fractions in Table 1.

Figure 1 shows the concentration dependencies of the reduced viscosities [η]_sp_/c for the OHLs and FWLs, and extrapolations to zero concentrations were completed using the Huggins equation (Equation (1)).

**Figure 2:**
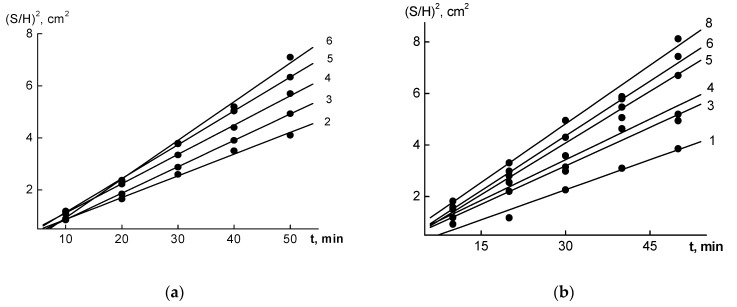
The time dependencies of the (S/H)^2^ dispersions of several fractions of the OHLs (**a**) and FWLs (**b**). The line numbers correspond to the numbers of the fractions in Table 1.

**Figure 3:**
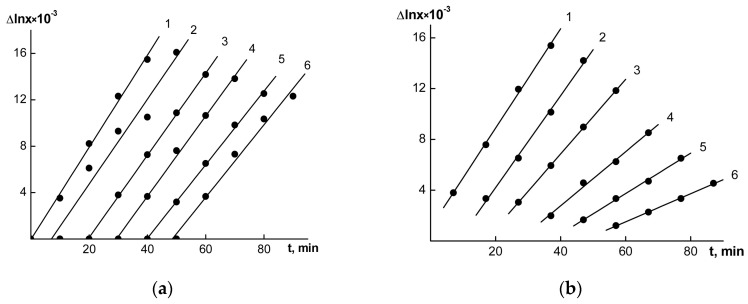
The relationships between Δlnx (x is the coordinate of the boundary maximum) and sedimentation time for the OHL (**a**) and FWL (**b**) fractions 1–6 in DMF. The numbers of the lines correspond to the numbers of the fractions in Table 1. Lines 2–6 were respectively shifted to the right along the time axis by 10 min (2), 20 min (3), 30 min (4), 40 min (5) and 50 min (6).

The resultant intrinsic viscosities [η] of the polymer fractions, along with the time dependencies of the diffusion boundary dispersions of the analyzed fractions, are shown in Figure 2, and the dependencies of Δlnx on Δt for the high molecular-weight fractions, as shown in Figure 3, allowed us to calculate the hydrodynamic characteristics of the OHL and FWL fractions. Table 1 shows the values of the MWs and the hydrodynamic characteristics of the fractions, which represented an array of raw data for the calculations of the fractal characteristics of the OHLs and FWLs.

Table 1 shows the values of the MWs and the hydrodynamic characteristics of the fractions, which represented an array of data used for the fractal analysis.

**Figure 4:**
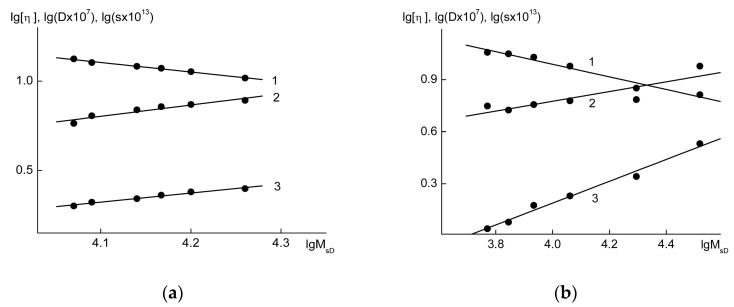
Dependencies of the translation diffusion coefficients (D) (1), intrinsic viscosity ([η]) (2), and sedimentation coefficients (s) (3) on the molecular weights (lgM_sD_) for the OHL (**a**) and FWL (**b**) samples.

The range of the MWs of the lignin fractions was not wide (from ~3 × 10^3^ to 30 × 10^3^), but it was sufficient for testing the applicability of the criteria of the fractality of the macromolecules (i.e., a scale invariance and a fractal dimension). The linear correlations between the MWs of the fractions and their coefficients of translational diffusion, sedimentation velocity, and characteristic viscosity ([η]) confirmed the scale invariances of the lignin fractions (Figure 4).

Macromolecular coils in Θ and good solvents can be described as stochastic fractal clusters. The analysis of fractal properties can help to retrieve additional information about the structure of a complicated macromolecule topology. However, a fractal analysis requires a set of several fractal parameters (dimensions) [12]. A polymer analysis uses the fractal dimension, d_S_, and the Renyi dimension, d_q_. One of the most informative parameters is the Hausdorff dimension, d_f_, which can be calculated by the following ratio (Equation (8)):

M ~ R^df^, (8)

where R is the radius of a macromolecule.

The fractal dimension d_f_ of a macromolecular coil in a solvent can be identified by its hydrodynamic characteristics using data on intrinsic viscosity, translational diffusion, and velocity sedimentation, as follows:

[η] = K_η_M^3/df−1^, (9)

D = K_D_M^1/df^, and (10)

s = K_S_M^(df−1)/df^. (11)

**Figure 5:**
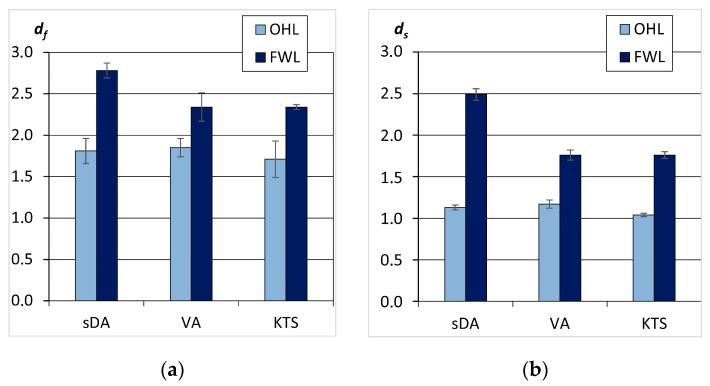
Values of d_f_ (**a**) and d_s_ (**b**) of the OHLs and FWLs according to results of the sedimentation-diffusion analysis (sDA), the viscosity analysis (VA), and the Kozlov–Temiraev–Sozaev method (KTS).

The data on the fractal dimension d_f_ of the OHL and FWL fractions are given in Figure 5. The measurements indicated that the d_f_ values of the oat husk lignins were between 1.71 and 1.85 while the d_f_ values of the FWLs were greater than 2.3. Thus, the studied conifer and herbaceous lignins belonged to different types of fractal objects.

Kozlov et al. [32] proposed an alternative method for estimating fractal dimensions that is worth considering for obtaining the fractal characteristics of macromolecules. The proposed method, as described by Equation (12), is based on the relationship between MW, the Huggins coefficient (k_H_), and the mass fractal dimension, as follows:

d_f_ = 3 lnMW/[lnMW + ln(7.14 k_H_ − 1) − lnK_η_ − ln k_H_]. (12)

Table 1 presents the results of the calculations of the fractal dimensions using the KTS method. The results showed very close d_f_ values for the factions, with small increases for higher MWs. However, the trends of the d_f_ values were accompanied by chaotic fluctuations in the Huggins coefficients (0.60–1.31). Analysis of these data using the fractal approach confirmed the reliability of the k_H_ measurements because the value of the fractal dimension using the KTS method was virtually the same as the d_f_ value found using the scaling parameters.

The d_f_ value of the FWLs was characteristic of Witten–Sander-type fractal objects. As noticed in [33], fractals of this type are theoretically formed by the diffusion-limited particle-cluster aggregation (DLA P-Cl), and the formation of physical fractal objects occurs in highly non-equilibrium conditions under the influence of dynamic self-organization. Additionally, regarding the details of the hydrodynamic behaviors and the conformational properties of the macromolecules of the fir lignins, this work confirmed a previous conclusion [13] where guaiacyl lignin macromolecules belong to a type of chaotically branched DLA P-Cl structures. The in vitro simulation and computer experiments on softwood lignin biosynthesis [13] supported this conclusion as well.

Based on the experimental estimates of d_f_ (Figure 5), the OHLs could be attributed to DLA Cl-Cl fractal objects. A simulation of DLA Cl-Cl processes and a study of the properties of these fractals were conducted by Meakin et al. [34] and Kolb et al. [35]. These models described aggregation in the three-dimensional space as follows: spherical particles of a certain size are randomly distributed in a cubic lattice, and then each of them move randomly within the lattice. The collision of two particles results in irreversible binding, with the formation of a dimer capable of further random movement. The aggregation of dimers with initial particles or two dimers leads to the formation of new clusters. The random movement and aggregation continue until a single large cluster is formed in the system. The results of the statistical processing of a large number of clusters permitted the determination of the scaling type, as follows (Equation (13)):

N ~ R^df^, (13)

where R is the cluster radius of the gyration and N is the number of particles in the cluster.

The fractal dimension of the clusters assembled from the monodispersed particles was equal to 1.78 ± 0.05. Polydispersed particle clusters are characterized by nearly the same fractal dimension. A previous work [36] assessed the impact of a series of the model parameters on cluster configuration, including the conditions and speed of diffusion, the probability of joining in a collision, and the reversibility of the process. The authors [12] showed that DLA Cl-Cl is the most suitable model for aerosol particles, and it can be used to characterize the fractal properties of linear polymers. The fractal dimension of DLA Cl-Cl aggregates is 1.75–2.0, which has been confirmed by an experimental work that grew clusters of different natures, including polymeric molecules. In particular, the authors of [12] concluded that the polymerization of polyacrylates with the formation of linear chains in selected circumstances is a process of cluster–cluster diffusion-limited aggregation.

In the context of the current discussion, important significance can be attached to the lignin biosynthesis modeling experiments in vitro and the study of the fractal properties of dehydropolymers (DHPs) (biosynthetic lignins). In a previous work [13], DHPs were synthesized via bulk and end-wise polymerization methods from ferulic acid (3-methoxy-4-hydroxy-cinnamic acid) in the presence of a horseradish peroxidase-hydrogen peroxide enzymatic system. The polymerization conditions under both synthesis variants were the same except for the monomer supply regime in the reaction zone, permitting comparisons with the classic models of fractal growth using DLA Cl-Cl and DLA P-Cl mechanisms. The results showed that the dynamics of the income monomer into the reaction zone were crucial for the structural organization of the DHP macromolecules. It was shown that the configuration of the macromolecules of bulk and end-wise dehydropolymers was variable, and the mass fractal dimension d_f_ for the bulk polymers was 2.62 ± 0.27, and for the end-wise dehydropolymers, d_f_ was 1.66 ± 0.16. These results argued strongly in favor of realistic hypotheses about the polyvariance of the topological and fractal structures of natural lignins. Continuing the discussion of the fractal structures of lignins from oat husks, it is appropriate to note the practical coincidence of the d_f_ values for lignins isolated from various species of grasses [9].

The mass fractal dimension characterizes the space occupancy degree, but not the connectivity, of elements that make up an object. According to Novikov and Kozlov [31], evaluation of the mass fractal dimension allows one to determine the conformation of macromolecules. At the same time, it is obvious that macromolecules have not only conformational fractal properties but also configuration properties, which are posed by mutual arrangements of topological elements such as knots, lateral branches, sub-chains, cross-linkers, cycles, etc. According to Mandelbrot, improvement in the descriptive accuracy of a complex structure requires an increase in the number of fractal dimensions [37]. To assess the connectivity of the structure elements (i.e., the macromolecule configuration), one can use the spectral or fracton dimension, d_s_, as proposed by [38], to characterize the oscillations of fractals. The fracton dimension relies on an equation of random walks on fractal lattices, and it can be computed using the following ratio (Equation (14)):

d_s_ = 2 d_f_/d_w_, (14)

where d_w_ = 2 + δ.

The value of the fractal dimension, d_w_, characterizes the particle random walk, which takes place on the neighboring monomeric units and can be determined by a scaling indicator of anomalous diffusion (δ). For any non-linearly configured macromolecules, the value of δ is always greater than zero. Accordingly, the value of the fracton dimension is greater than unity. A feature of the fracton dimension is the independence of its values from the method of implementation of the fractal object in Euclidean space. In other words, the fracton dimension, unlike the fractal dimension of the Hausdorff type, is determined mainly by the topological structure of an object. Therefore, solving the problem of the structural organization of new macromolecules requires scoring the fracton dimension of ds. According to Vilgis [39], the d_s_ of a macromolecule in solvent can be calculated using Equation (15):

d_f_ = d_s_(E + 2)/(d_s_ + 2), (15)

where E is the dimension of Euclidean space.

This parameter characterizes the topologies of macromolecules because it is determined by the levels of connectivity of the monomeric system components. For a linear macromolecule, the value of the fracton dimension is equal to one. For branched molecules, the value of d_s_ is greater than one. According to available data [38], the value of d_s_ is equal to 1.33 for critical percolation clusters, and it is more than 1.4 for randomly branched fractals of the Witten–Sander type. According to our own studies, the value of ds depends on a number of the parameters of the DLA P-Cl model, and it can reach a magnitude of 2.3.

As can be seen from Figure 5, the d_s_ values for the OHLs (herbaceous lignins) differed very slightly from one, whereas the d_s_ values for the FWLs (wood lignins) reached 2.5 according to sedimentation and diffusion analyses, with a value of 1.7 according for the viscometry analysis. Therefore, if one relies on the ds index, at first approximation, the topologies of FWL macromolecules are characterized as highly branched, and the topologies of OHLs are characterized as linear. In principle, the same conclusion can be approached by using the traditional analysis of scaling indexes, which are calculated according to Mark–Kuhn–Houwink (M-K-H) relationships. In particular, the hydrodynamic behaviors of the oat lignins DMF system can be described by the following indexes: M-K-H: a = 0.62, b = −0.52, and c = 0.48, respectively.

Thus, the fractal analysis suggested that the lignin macromolecules that are components of softwood fir can be characterized by sufficiently strongly branched structures. Within the fractal concept, this type of lignin must be treated as an object that is formed according to the regularities of the diffusion-limited aggregation of the particle-cluster type, and the lignin macromolecules of the OHLs belong to the DLA Cl-Cl class of fractals, with a predominance of characteristics common to a linear configuration.

It should be noted that the values of the fractal dimensions of the OHLs and FWLs established in this work were consistent with the results of previous hydrodynamic studies that were performed for lignins of other gymnosperms and grassy plants [40,41]. Thus, we calculated the values of the fractal dimension (d_f_) for Björkman lignins (LBs) of pine wood according to the hydrodynamic results provided in the work of Pavlov G.M. and collaborators [42]. The calculations showed that the fractal dimensions of the pine lignins in the LB-DMSO system were 2.32 according to the diffusion experiments and 2.40 according to the viscometry data. For spruce lignins (based on sedimentation experiments), the d_f_ calculations [21] provided a value of 2.44. Thus, mutually consistent results have been obtained for lignins from various coniferous plants.

Somewhat contradictory results have been obtained for the lignins of grassy plants. A study of the topologies of the macromolecules of wheat, rye, and barley lignins showed that these biopolymers, similar to the OHL sample, are linear polymers with a fractal dimension of approximately 1.7 [9]. However, lignins isolated from another cereal plant, bamboo, turned out to be randomly branched polymers [43]. Calculations of the fractal dimension (d_f_ = 2.2–2.4) and branching density according to the Kogan–Gandelsman–Budtov model have shown that bamboo lignins, from the point of view of topological structures, are close to lignins of coniferous wood species.

Extremely interesting results have been obtained in the study of the topological structures of other taxonomic classes of herbaceous plants. For example, lignins isolated from the roots of *Rhodiola rosea* L. were assigned to the universal class of star-shaped macromolecular compounds (d_f_ = 1.6) [44]. The authors of [45] showed the hydrodynamic properties and topologies of pepper lignins from the stems of *Serratula coronata* L., and the study’s data showed that the behaviors of these herbaceous lignins corresponded to hyperbranched polymers. Thus, lignins show great diversity in their the topological structures, which makes it impossible to make an a priori prediction for the structures of lignins of different taxonomic origins. In this regard, it is necessary to actively continue research on the macromolecular properties of various lignins, especially grassy plants, and this can provide a solution to the problem of using lignins in various fields, including biotechnology and biomedicine.

In the discussion above, we have not considered the subject of the relationship between molecular mass scaling and established methods of molecular hydrodynamics, along with the phenomena of analogous polymer and scale invariances. In addition, our discussion about the results did not go beyond a perception of the studied sites as monofractals for which the principle of self-similarity is unambiguous. However, the fractal analysis of the studied biopolymer fractions (Table 1) detected the presence of a certain distribution for the values of d_s_ and d_f_. Future research may be warranted to understand the structural organizations of lignin macromolecules in association with ideas about multifractals. An interpretation of the term multifractal as applied to polymer molecules is reviewed in [5].

## 4. Conclusions

The analysis of scaling dependencies in polymer–solvent systems and the determination of the fractal dimensions of lignins from oat husks (*Avena sativa* L.) and fir trees (*Abies sibirica* Ledeb.) have allowed for the establishment of the features of the topological structures of the lignins. This study was based on the experimental methods of velocity sedimentation, capillary viscometry, and translational diffusion, which provide a direct quantitative assessment of the coefficients of the rotational and translational friction of the macromolecules in diluted solutions. The measurements indicated that the fractal dimensions (d_f_) of guaiacyl-syringyl lignins (the OHLs) were between 1.71 and 1.85 while the d_f_ values of the FWLs, the typical guaiacyl lignins, were substantially higher (~2.3).

To establish the features of the topologies of the studied lignins, a new and practical approach to the analysis of hydrodynamic data for the “polymer-solvent” system was implemented based on the identification of the relationships between the conformational, scaling, and fractal characteristics of lignins. This approach led to the important conclusion that the lignin macromolecules of oat husks belong to the DLA Cl-Cl class of fractals of the Meakin–Kolb type and the softwood lignins of fir trees exhibit branched topological structures and belong to the DLA P-Cl class of fractals of the Witten–Sander type.

These new results include the conclusion that oat husk lignins belongs to the class of linear macromolecular compounds. For the first time, the reliability of experimental results on the chaotic nature of fluctuations in the Huggins coefficients for narrow fractions of the studied lignins was confirmed, as evidenced by the relationship between their values and the fractal indices.

The results of this work indicate the possibility of the targeted search of lignins of a certain botanical origin in order to create new polymeric lignin-based materials. The obtained experimental data can be used for the further development of the topotaxonomic classifications of native lignins.

The main points of this research are as follows:

- the lignins of *Avena sativa* L. belong to the class of linear polymers
- the macromolecules of *Avena sativa* L. lignins in solution are fractal objects of the Mikin–Kolb type
- the macromolecules of the lignins of the fir tree *Abies sibirica* Ledeb. are fractal objects of the Witten–Sander type

## Figures and Tables

**Table 1:** Hydrodynamic and fractal characteristics of the OHLs and FWLs.

Fraction	M_Dη_ × 10^–3^	[η] (cm^3^/g)	D × 10^7^ (cm^2^/c)	M_sD_ × 10^–3^	s × 10^13^ ©	k_H_	d_f_	d_s_
Lignins from oat husks (OHLs)								
1	18.9	7.8	10.4	18.3	2.5	0.63	1.75	1.07
2	15.6	7.4	11.3	16.0	2.4	0.79	1.73	1.06
3	14.0	7.2	11.8	14.7	2.3	0.86	1.72	1.05
4	13.6	6.9	12.1	13.8	2.2	0.77	1.72	1.05
5	12.7	6.4	12.7	12.4	2.1	1.27	1.71	1.04
6	12.2	5.8	13.3	11.7	2.0	0.83	1.71	1.04
7	9.1	5.5	14.9	-	-	0.72	1.69	1.02
8	7.7	5.1	16.2	-	-	0.63	1.68	1.01
Lignins from fir wood (FWLs)								
1	22.0	9.5	6.5	33.0	3.4	1.26	2.37	1.80
2	26.3	6.1	7.1	19.7	2.2	1.31	2.38	1.82
3	11.2	6.0	9.5	11.5	1.7	0.70	2.35	1.77
4	8.2	5.7	10.7	8.6	1.5	0.69	2.34	1.76
5	7.7	5.3	11.2	7.0	1.2	1.02	2.32	1.73
6	6.9	5.6	11.4	5.9	1.1	0.65	2.33	1.75
7	6.0	5.1	12.3	-	-	0.84	2.31	1.72
8	3.9	4.8	14.5	-	-	0.60	2.30	1.70

## Data Availability

The raw data required to produce these findings can be shared by the authors upon request. Readers are encouraged to communicate with the corresponding author for more information.
